# Association between the total amount of electromagnetic cortical neuronal activity and a decline in motivation

**DOI:** 10.14814/phy2.15028

**Published:** 2021-09-23

**Authors:** Akira Ishii, Takashi Matsuo, Takahiro Yoshikawa

**Affiliations:** ^1^ Department of Sports Medicine Osaka City University Graduate School of Medicine Osaka Japan

**Keywords:** biological alarm; fatigue, electromagnetic neural activity; magnetoencephalography, fatigue sensation, motivation

## Abstract

In situations involving fatigue, the increase in fatigue levels and the apparent decrease in motivation levels are thought to suppress mental and physical performance to avoid disrupting homeostasis and aid recovery; however, the ultimate source of information on which the brain depends to perceive fatigue and/or a loss of motivation for protection remains unknown. In this study, we found that, as assessed by magnetoencephalography, electromagnetic cortical neuronal activity while performing cognitive tasks was associated with a decrease in motivation caused by the tasks in healthy participants, suggesting the possibility that the brain utilizes information that reflects the invested amount of neural activity to suppress performance. To our knowledge, this is the first report to provide clues for the missing link between neural investments and the resulting activation of the biological alarms that suppress performance.

## Graphical abstract

Electromagnetic cortical neuronal activity while performing cognitive tasks was associated with a decrease in motivation caused by the tasks in healthy participants, suggesting the possibility that the brain utilizes information that reflects the invested amount of neural activity to suppress performance in the situation of fatigue.

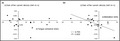

## 1 INTRODUCTION

Fatigue has been defined in various ways depending on the aspect of fatigue focused on by each researcher. For example, fatigue has been defined as a decline in either the ability to perform or the efficiency of performing mental and/or physical activities caused by excessive mental or physical activity or disease that is often accompanied by a peculiar sense of discomfort, a desire to rest, and a decline in motivation; this is referred to as fatigue sensation (Kitani, 2011).

Fatigue sensation seems not to be merely the resultant sensation caused by mental and/or physical activity: Fatigue sensation is thought to be an essential biological alarm that urges individuals to rest to prevent a disruption in homeostasis and recover from fatigue (Boksem et al., 2006; Ishii et al., 2014; Tanaka & Watanabe, 2010). It has been proposed that the impairments of the parts of the body and/or the brain caused by mental and/or physical activity are not the only cause of fatigue and that there exist regulatory neural mechanisms which are to suppress performance based on the perceived level of increased fatigue sensation and/or that of decreased motivation caused by the activity, leading to the decline in mental and/or physical performance (i.e., fatigue) (Ishii et al., 2014; Tanaka et al., 2013; Tanaka & Watanabe, 2010, 2012): In situations involving fatigue, an increase in the subjective level of fatigue sensation and/or decrease in the level of motivation are thought to suppress mental and physical performance, avoiding further cellular damage caused by oxidative damage, excessive generation of cytokines, and energy deficiency in the parts of the body engaged in the activity (Ishii et al., 2014; Tanaka et al., 2013). Therefore, the perception of fatigue sensation and motivation is the important component in the neural mechanisms of fatigue; however, the ultimate source of the information on which the brain depends to perceive fatigue sensation and/or a loss of motivation for protection remains unknown. Gaining a better understanding of the source of this information is fundamental for clarifying the neural mechanisms underlying fatigue.

In the case of physical fatigue, the afferent input from peripheral tissues such as the small‐diameter muscle afferents may be a source of this information (Tanaka & Watanabe, 2012). However, the muscles and other peripheral tissues are not the only components of the body excessively activated, and thereby protected, while performing an activity. The neurons that contribute to the preparation and execution of physical activities may also be damaged by excessive performance. In the case of mental fatigue, which is a type of fatigue caused by mental activities such as cognitive tasks, the neurons engaged in the activity are also in danger of overactivation, so it is necessary to suppress performance to protect these neurons. Therefore, we hypothesized that the brain utilizes the information associated with the amount of neural activity (i.e., the cumulative amount of the neural activity invested in physical and/or mental activities) to avoid neural damage caused by excessive physical and mental activities. However, it is difficult to measure the activity of all neurons in the brain precisely in humans. Therefore, we decided to calculate the total amount of cortical neuronal activity cumulated over the performance of cognitive tasks, which can be approximated noninvasively using magnetoencephalography (MEG), with the assumption that the total amount of cortical neuronal activity in the brain would reflect the overall number of adverse events caused in the brain more accurately than the amount of neural activity in specific brain regions.

In this study, the participants performed two types of cognitive tasks, and the association between the differences in the alterations of fatigue and motivation levels and in the total amount of cortical electromagnetic activity cumulated over each task were examined. The current density in the cortical area caused by each single trial was assessed by a minimum norm estimate analysis of the MEG data, and the cumulative total amount of neuronal activity in the cortex throughout each task was calculated as the summation of “the summation of the current density within each trial” across all trials in each task. The norm of the current density was used for this calculation and thus, both evoked and induced neural activity contributed to the estimates of the cortical electromagnetic activity in our present study. Since it has been reported that the electromagnetic neural activity in alpha and beta bands (i.e., 8–25 Hz) contribute to cognitive processes (Grabner & De Smedt, 2011; Jost, Beinhoff, et al., 2004; Jost, Hennighausen, et al., 2004; Keil et al., 2006; Ku et al., 2010; Pfurtscheller & Lopes da Silva, 1999; Wei et al., 1998) and limit the frequency range to eliminate the signals that do not originate from neural activity (Herrmann et al., 2014; Tremmel et al., 2019; Yuval‐Greenberg & Deouell, 2011), we analyzed MEG signals in the 8–25 Hz frequency band.

## 2 EXPERIMENTAL PROCEDURE

### 2.1 Participants

Twenty‐six healthy male volunteers aged 21.5 ± 1.7 years (mean ± standard deviation [*SD*]) participated in this study. All participants were confirmed to be right‐handed according to the Edinburgh Handedness Inventory (Oldfield, 1971). None of the participants reported having any health problems such as neural disorders, brain injury, or a history of mental illness. Current smokers and individuals taking chronic medications that affect the central nervous system were excluded. The Ethics Committee of Osaka City University approved the study protocol (approval No. 4227). Each participant provided written informed consent to participate in this study in accordance with the Declaration of Helsinki and the ethical guidelines for medical and health research involving human subjects in Japan (Ministry of Education, Culture, Sports, Science, and Technology and Ministry of Health, Labour and Welfare). The participants were undergraduate students of Osaka City University. The experimenters were not in charge of their education and recognition of credit (i.e., they participated without course credit). They were paid 18,000 yen (about $180) for the participation.

### 2.2 Tasks

**FIGURE 1:**
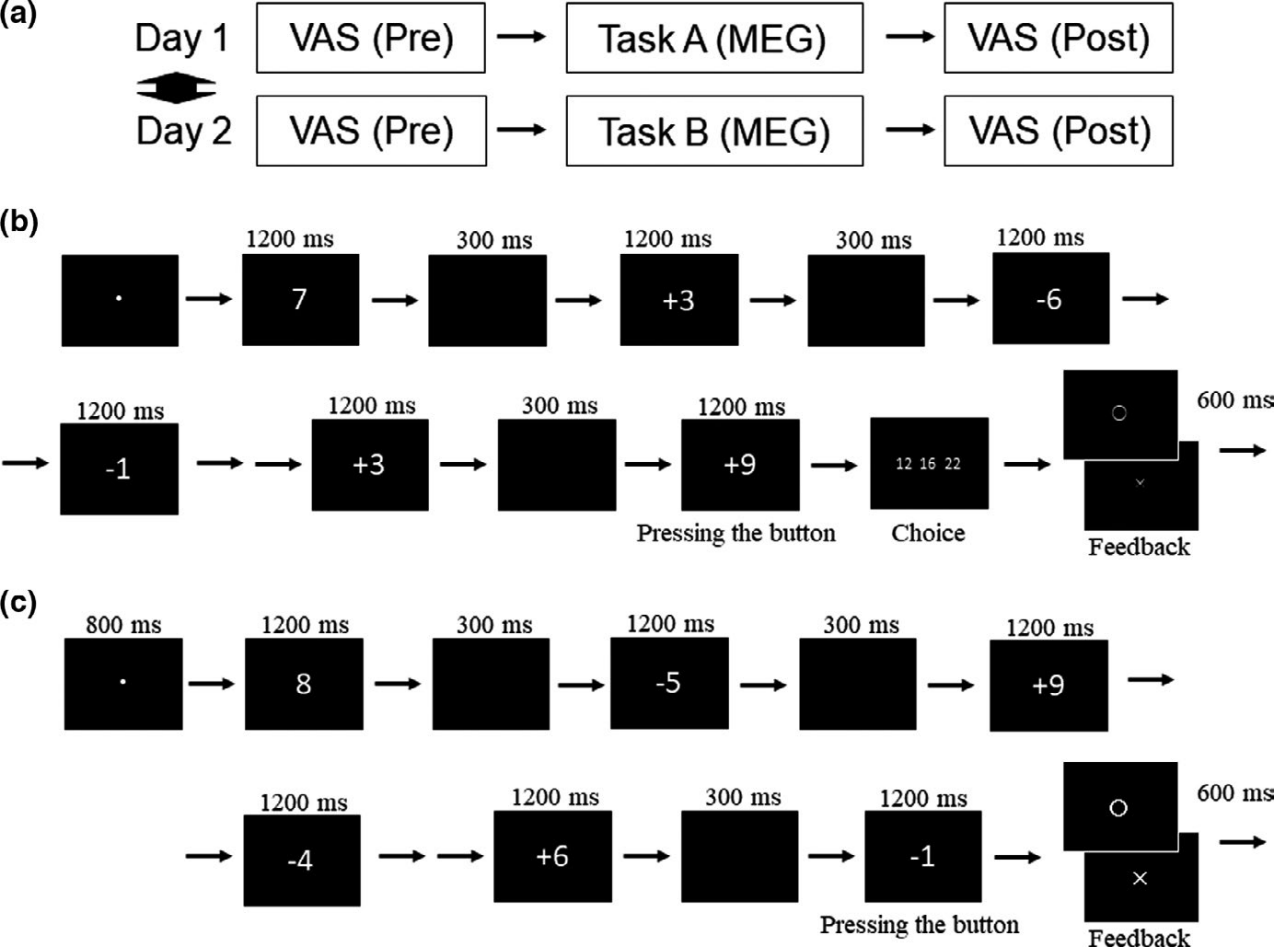
Experimental procedure. (a) The participant performed two types of cognitive tasks (i.e., tasks A and B) on a different day in a two‐crossover design. (b) Task A was a mental calculation task: following the presentation of one‐digit natural number for 1200 ms, a one‐digit natural number in combination with an operator (i.e., “+” or “–”) was sequentially presented for 1200 ms every 1500 ms. The participants were asked to continue the mental calculation of the numbers presented as long as they could. They were asked to press a button with their right index finger when they could no longer continue calculating, and then to select the answer for the calculation corresponding to just one presentation before the one in which they pressed the button by pressing the left, middle, or right button with their right index, middle, or fourth finger, respectively. After receiving feedback informing them of whether their choice was correct, a new set of mental calculation trials began. (c) The presentation of task B was similar to that of task A; however, in task B, the participants were asked to press a button with their right index finger only when the combination of a presented number and operator was “–1,” and not to calculate the sequential numbers presented mentally. Task A and task B were programmed to last for 20 min. VAS, visual analogue scales for fatigue sensation and motivation

Each participant performed two types of cognitive tasks (i.e., task A and task B) on a different day in a two‐crossover design (Figure 1a). Task A was a mental calculation task: following the presentation of one‐digit natural number for 1200 ms, a one‐digit natural number in combination with an operator (i.e., “+” or “–”) was sequentially presented for 1200 ms every 1500 ms (Figure 1b). The participants were asked to continue the mental calculation of the numbers presented as long as they could. They were asked to press a button with their right index finger when they could no longer continue calculating, and then to select the answer for the calculation corresponding to just one presentation before the one in which they pressed the button by pressing the left, middle, or right button with their right index, middle, or fourth finger, respectively. After receiving feedback informing them of whether their choice was correct, a new set of mental calculation trials began. The number and operator in each presentation were selected at random; however, with the aim of controlling the difficulty of the task so that the participants could continue calculations for longer periods without a button press, combinations that caused the result of the calculation to be negative were replaced by another set for which the result would not be negative. The presentation of task B was similar to that of task A (i.e., the number and operator in each presentation were selected at random); however, in task B, the participants were asked to press a button with their right index finger only when the combination of a presented number and operator was “–1,” and not to calculate the sequential numbers presented mentally (Figure 1c). Task A and task B were programmed to last for 20 min.

The subjective levels of fatigue sensation and task motivation were assessed just before and after each task by a visual analog scale (VAS) of 100 mm. The VAS was used to assess the levels of fatigue sensation and motivation because the procedure is simple and can be completed in a short time: It is important to collect the data regarding the levels of fatigue sensation and motivation immediately after the tasks to avoid recovery from fatigue. The VAS has been used to assess the levels of fatigue sensation and task motivation (for example Fritz & O'Connor, 2016; Kleih et al., 2010; Lee et al., 1991; Mizuno et al., 2020; Tajima et al., 2010; Wykowska et al., 2013).

### 2.3 MEG recording

The neural activity was recorded from the start to the end of each task (i.e., task A and task B) using a 160‐channel whole‐head‐type MEG system (MEG Vision; Yokogawa Electric Corporation) with a magnetic field resolution of 4 fT/Hz^1/2^ in the white noise region. The sensor and reference coils were gradiometers with a 15.5‐mm diameter and 50‐mm baseline, and the two coils were separated by 23 mm. The sampling rate was 1000 Hz, and the obtained data were high‐pass filtered at 0.3 Hz. Empty room recordings for 5 min were carried out to estimate a noise covariance matrix immediately before or after each experiment.

### 2.4 Magnetic resonance imaging (MRI)

Anatomical magnetic resonance imaging (MRI) was performed using a Philips Achieva 3.0 TX scanner (Royal Philips Electronics) to permit the registration of magnetic source locations with their respective anatomical locations. Five markers (Medtronic Surgical Navigation Technologies, Inc.) were attached to the scalp (i.e., two markers 10 mm in front of the left and right tragi, one marker 35 mm above the nasion, and two markers 40 mm to either side of the marker above the nasion). The MEG data were co‐registered to the MRI images using information obtained from these markers and the MEG localization coils. Cortical automated segmentation of the MR images was performed using FreeSurfer software, which is documented and freely available for download online (http://surfer.nmr.mgh.harvard.edu/).

### 2.5 MEG analyses

The MEG data from 10 participants were excluded from the analyses and therefore, the data from 16 participants were analyzed: the data from four participants were excluded from the analyses because of software issues, the data from the other five participants were excluded because their MEG data included at least one segment of epochs with artifacts, identified visually as describe below, longer than 3 min, and the data from one participant were excluded because of the failure in the corresponding empty room recording.

**FIGURE 2:**
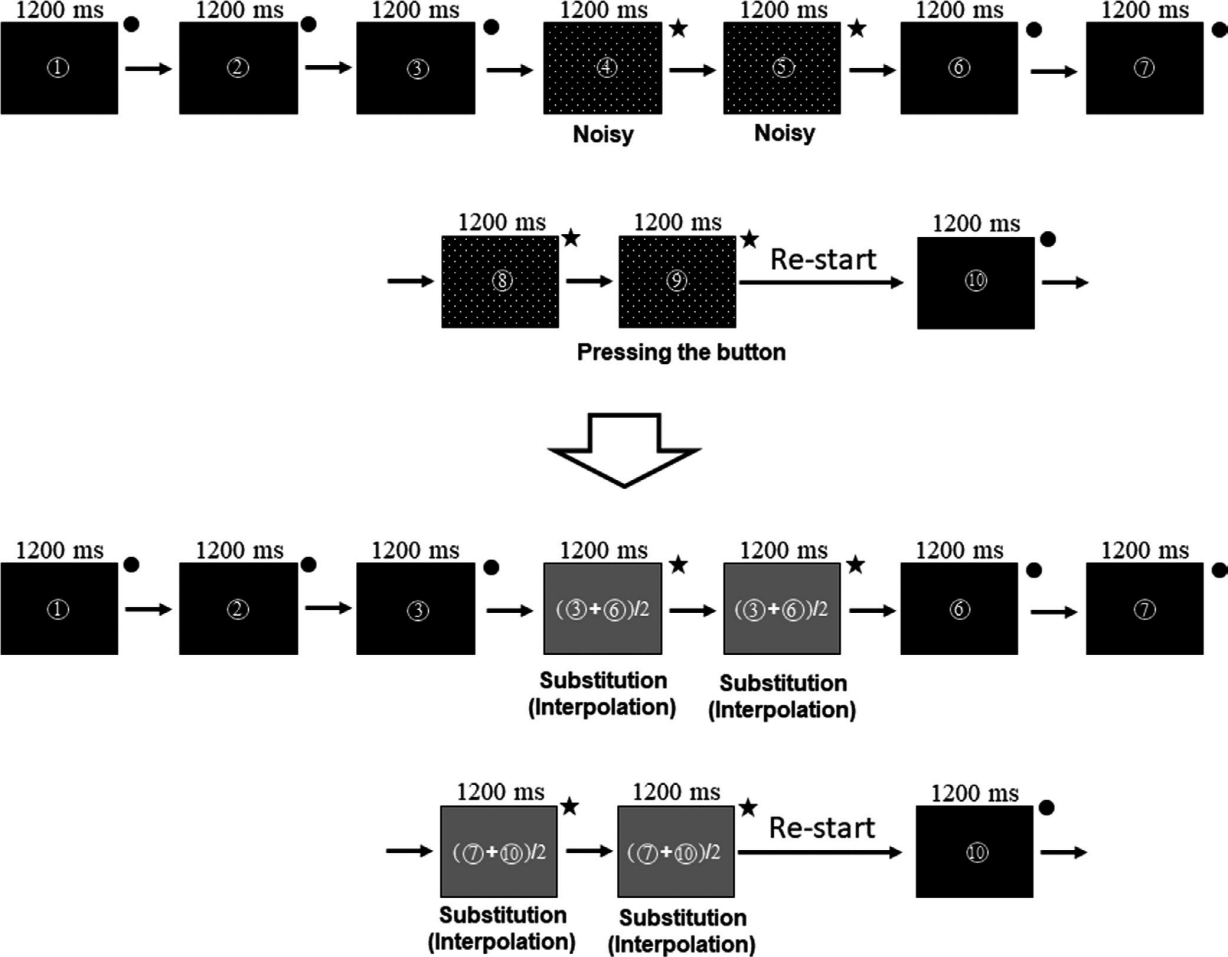
Epochs for which the original neural activity values were interpolated. The presentations with closed circles correspond to the epochs for which the original neural activity values were used, and those with stars correspond to the epochs for which the original neural activity values were replaced by the mean value of the epochs with closed circles just before and after the segment of epochs with stars

Magnetic noise from outside the magnetically shielded room was eliminated by subtracting the data obtained from the reference coils using a software program (MEG 160; Yokogawa Electric Corporation). The MEG data corresponding to the presentation of each number (or the combination of a number and an operator) were epoched. The epochs including artifacts were visually identified, and in the later analyses, the value of the sum of the current density within each epoch in these contaminated epochs was replaced by the mean of the values just before and after the contaminated segment of epochs (Figure 2). In addition to the contaminated epochs, the epochs during which the button was pressed and the epochs at one presentation before the epoch with the button press were also identified, and the value of the sums of the current densities in these epochs was replaced by the mean of the values just before and after these epoch segments to exclude the possible inclusion of any myogenic artifacts caused by pressing the button (Figure 2).

The MEG data were exported from the recording software (MEG 160; Yokogawa Electric Corporation) in a unique data format and converted to the format used by Brainstorm software (Tadel et al., 2011). Then, the MEG data were band‐pass filtered at 8–25 Hz using a finite impulse response filtering method. The source estimation for each single epoch was performed applying a minimum norm algorithm (Baillet et al., 2001) with a realistic head model for each participant, and a noise covariance matrix was estimated using the noise‐free segment of the empty‐room recordings for 19 s (Tadel et al., 2011). The current density was calculated without orientational constraints to avoid the underestimation of the neural activity (i.e., the “Unconstraint” option in Brainstorm software). To calculate the sum of the cortical electromagnetic activity within each single epoch, the norm of the current density within the time range of the single epoch was integrated across time (i.e., 0–1200 ms) at each cortical region, and then the integrated values at each cortical region were multiplied by the number of vertices in the corresponding brain region to estimate the sum of the activity within the brain region rather than the averaged activity. Finally, the resulting values for each brain region were summed across the brain and then further across trials (i.e., all the epochs in each task). Segmentation of the cortical regions of each participant was performed based on the Destrieux atlas (Destrieux et al., 2010), and thus, the values at 148 cortical regions (i.e., 74 cortical regions per hemisphere) were calculated and summed.

### 2.6 Statistical analysis

The normality of the data regarding the subjective levels of fatigue and motivation and the estimated sum of the cortical electromagnetic activity were confirmed using the Kolmogorov–Smirnov test. All values are presented as mean ± *SD* unless otherwise stated. The relationships between the differences in the alterations of fatigue and motivation levels and the total amounts of cortical activity between tasks were examined using Pearson's correlation coefficient. To control for the effects caused by the difference in the numbers of button presses and trials in each task, multiple regression analyses were performed with the difference of the alteration in the level of motivation between tasks as a dependent variable and that of the total amount of cortical activity between tasks as the independent variable. The normality of the residuals of these multiple regression analyses was confirmed using the Kolmogorov–Smirnov test. All *p* values were two‐tailed, and values <0.05 were considered statistically significant.

## 3 RESULTS

### 3.1 Subjective levels of fatigue and motivation assessed just before and after task A and Task B

The participants performed a demanding cognitive task (i.e., mental calculation task) and an undemanding cognitive task (i.e., simple button‐press task) (task A and task B, respectively). The subjective levels of fatigue and motivation just before and after each task (i.e., task A and task B) assessed using VAS are shown in Table 1. There was no association between the alteration of the level of fatigue sensation and that of motivation (*r* = 0.220, *p *= 0.414).

**TABLE 1:** Subjective levels of fatigue and motivation in each task

	Task A		Task B	
	Before	After	Before	After
Fatigue (mm)	22.5 ± 12.5	35.6 ± 13.1	25.0 ± 22.4	31.3 ± 17.8
Motivation (mm)	54.8 ± 18.3	46.9 ± 15.3	53.3 ± 19.8	48.8 ± 17.5

Data are presented as mean ± standard deviation.

### 3.2 Numbers of trials and button presses performed in task A and task B

The numbers of trials and button presses performed in each task (i.e., task A and task B) are shown in Table 2.

**TABLE 2:** Numbers of trials and button presses in each task

	Task A	Task B
Number of trials	694.3 ± 18.7	706.1 ± 3.9
Number of button presses	23.2 ± 14.4	38.5 ± 5.5

Data are presented as mean ± standard deviation.

### 3.3 Electromagnetic cortical activity invested in task A and task B

The current density in the cortical area caused by each single trial was assessed by a minimum norm estimate analysis of the MEG data, and the total amount of neuronal activity in the cortex cumulated over each task was calculated as the summation of “the summation of the current density within each trial” across all trials in each task. The norm of the current density was used for this calculation. The electromagnetic neural activity in alpha and beta bands (i.e., 8–25 Hz) was assed and The sum of the electromagnetic activity in the cortex (i.e., the total cortical neuronal activity integrated in time) for each task (i.e., task A and task B) is shown in Table 3.

**TABLE 3:** Accumulation of cortical neuronal activity throughout each task

	Task A	Task B
Total cortical neuronal activity (mA∙m∙s)	0.0495 ± 0.00711	0.0521 ± 0.00847

Data are presented as mean ± standard deviation.

### 3.4 Relationships between total cortical neuronal activity and subjective levels of fatigue and motivation

**FIGURE 3:**
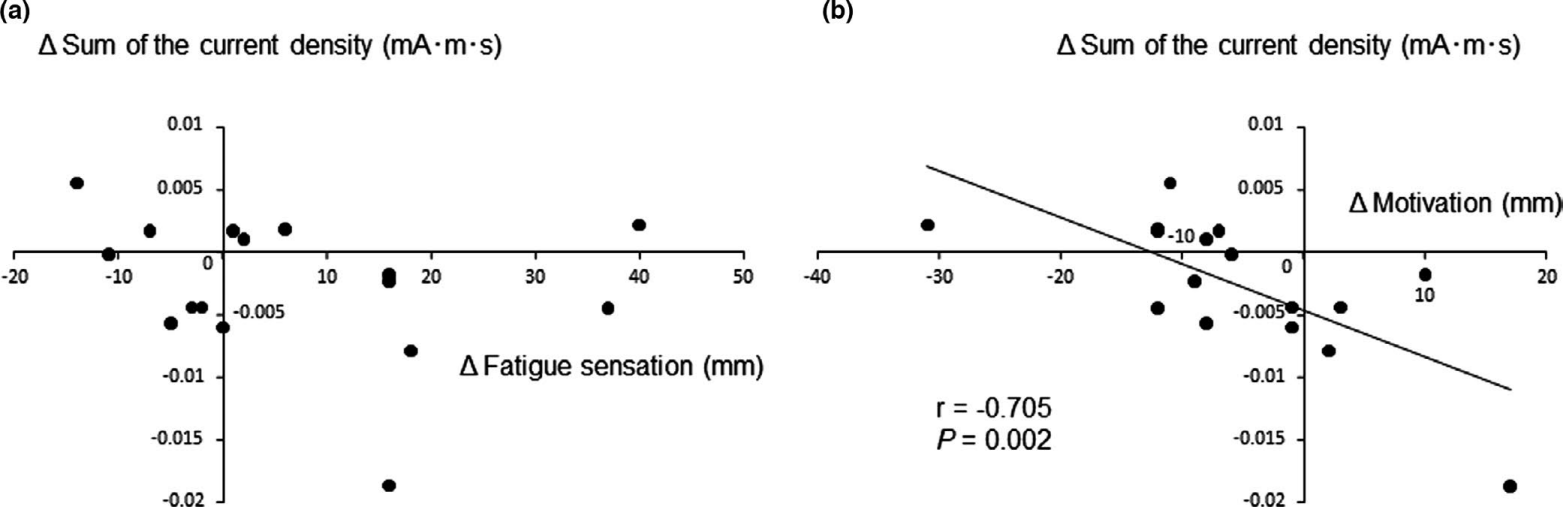
Relationships between the total cortical neuronal activity and subjective levels of fatigue and motivation. (a) Scatterplot of the difference in the total amount of cortical neuronal activity between tasks versus the difference in the alteration of the fatigue levels between tasks. (b) Scatterplot of the difference in the total amount of cortical neuronal activity between tasks versus the difference in the alteration of motivation levels between tasks. The linear regression line, Pearson's correlation coefficient, and *p* value are shown

No association was observed between the difference in the alteration of fatigue levels and that in the total amount of cortical activity between tasks (*r* = –0.238, *p *= 0.375; Figure 3a). The difference in the alteration of motivation levels between tasks was negatively associated with that in the total amount of cortical activity between tasks (*r *= –0.705, *p *= 0.002; Figure 3b). We intended to evaluate the sum of electromagnetic neuronal activity throughout the cognitive tasks (i.e., calculating the cumulative electromagnetic neuronal activity from the start to the end of each task) because we hypothesized that the brain utilizes the information associated with the amount of neural activity invested in physical and mental activities in order to avoid neural damage. Therefore, the correction of the number of trials was not necessary in our present study; however, since we wanted to exclude the possibility that our finding that the difference in the alteration of motivation levels between tasks was associated with that in the total amount of cortical activity between tasks was caused by unknown confounding mechanisms related to the number of button press performed by the participants, we performed additional multiple regression analyses to show that the association between the difference of the alteration in motivation levels between tasks and that of the total amount of cortical activity between tasks remained even after controlling for the difference in the numbers of trials (i.e., the difference of the number of trials was caused by the difference of the number of button press) and button presses in each task. After performing the multiple regression analyses, the association between the difference of the alteration in motivation levels between tasks and that of the total amount of cortical activity between tasks remained, even after controlling for the difference in the numbers of trials and button presses in each task (Table 4). The correlation between the alteration of the level of motivation and that in the total amount of cortical neuronal activity showed a tendency to be stronger than the correlation between the alteration of the level of fatigue sensation and that in the total amount of cortical neuronal activity (*p *= 0.0788, Hotelling's method to compare correlation coefficients; Hotelling, 1940).

**TABLE 4:** Results of multiple regression analyses with the difference in the alteration of motivation levels between tasks as the dependent variable

Independent variables	β	*r*
Model 1		
Total neural activity	–0.62*	–0.71**
Number of trials	–0.13	–0.54*
*R* ^2^	0.51	
Adjusted R^2^	0.43	
*N*	16	
Model 2		
Total neural activity	–0.57*	–0.71**
Number of button presses	0.26	0.56*
*R* ^2^	0.55	
Adjusted *R* ^2^	0.48	
*N*	16	

β, standard partial regression coefficient; *r*, correlation coefficient; *R* ^2^, coefficient of determination; *N*, the number of the subjects.

* *p* < 0.05, **p < 0.01.

### 3.5 Subanalysis of the correlations between regional cortical electromagnetic activity and subjective motivation levels

An examination of the correlations between the difference in the accumulation of cortical electromagnetic activity in 148 brain regions anatomically parcellated based on the Destrieux atlas (Destrieux et al., 2010) and that in the alteration of motivation levels between tasks revealed that no brain regions met the statistical threshold after correcting for multiple testing using the Benjamini–Hochberg method, which controls the false discovery rate.

## 4 DISCUSSION

In this study, the participants performed two types of cognitive tasks (i.e., a mental and a non‐mental arithmetic task), and the total cortical neuronal activity throughout the tasks was assessed by way of a minimum norm estimate of the MEG data. While no association was observed between the difference in fatigue level and in that of the total amount of cortical neuronal activity between tasks, a negative association was found between the difference in the alteration of motivation levels and that in the total amount of cortical neuronal activity between tasks. Although the number of trials performed in each task differed depending on each participant's response (i.e., the number of times each participant pressed the button in each task), this association remained apparent even after controlling for differences in the numbers of trials and button presses in each task as control variables in the multiple regression analyses, with the difference in the alteration of the motivation level between tasks as the dependent variable and that in the total amount of cortical neuronal activity between tasks as the independent variable. Therefore, the association between the difference in the alteration of motivation levels and that in the total amount of cortical activity between tasks was not caused by differences in the numbers of trials or button presses, which depended on each participant's response.

There have been reports on the alterations of the neural activity caused by performing cognitive tasks: For example, it has been reported that, during the performance of a demanding cognitive task, the decline of the neural activity related to performing the task and the emergence of the compensatory neural activity were observed (Babu Henry Samuel et al., 2019) and that the alterations of the connectivity between brain regions (Borragan et al., 2019) or the alterations of the network configuration of the brain (Kitzbichler et al., 2011) were related to performing cognitive tasks. In addition, it has been shown that a parameter describing the network configuration of the brain assessed during 0‐back and 2‐back tasks explained the task performance of the participants (Duman et al., 2019). The neural mechanism of mental calculation has also been investigated in terms of the processing stage of mental arithmetic (Pinheiro‐Chagas et al., 2019). Although alterations of neural activity caused by performing cognitive tasks have been intensively investigated as mentioned above, there have been no previous reports that focused on the relationships between the subjective level of fatigue and/or motivation and the alteration of the neural activity in terms of the sum of the cortical neural activity cumulated over cognitive task (i.e., the amount of the cortical neural activity integrated over time) invested to perform the task.

Since the norm of the current density (i.e., the value is always positive) in the cortical area caused by a single trial was integrated within the time range of each trial (i.e., 0–1200 ms) and the resulting integrated current density within each single trial was summed across trials, the total electromagnetic cortical neuronal activity assessed in this study included the neural activity corresponding to both evoked (i.e., activities that were time‐ and phase‐locked to the stimulus onset) and induced (i.e., activities that were time‐ but not phase‐locked to the stimulus onset) activities (i.e., total activity) (Herrmann et al., 2014; Sauseng et al., 2007). Evoked and induced neuronal activities are usually assessed as values relative to those at baseline segments which are the parts of the MEG data recorded over the task trials; however, we used the empty‐room recordings to estimate noise covariance rather than using some segments of the MEG recording during which tasks were performed because we focused on evaluating the total amount of electromagnetic cortical neuronal activity across the cognitive tasks rather than relative increases from the baseline segments of the MEG data recorded over the task trials (i.e., the neural activity such that exists in the segments would be attenuated in the source reconstruction if the baseline segments of the MEG data recorded over the task trials are used to estimate noise covariate). Therefore, although our estimates of the cortical neuronal activity throughout the tasks included the neural activity corresponding to the induced activities, the amount of the activity was not assessed in terms of relative change in baseline segments of the MEG data recorded over task trials as previous studies: Since it has been reported that whether induced activities caused by a cognitive task increase or decrease in relation to baseline segments of the MEG data recorded over task trials depends on the baseline activity level and that the baseline activity, which is included in our estimates of the total cortical neuronal activity, may also play a functional role in upcoming information processing (Min et al., 2007), our estimates of total cortical neuronal activity are thought to reflect the neuronal effort invested in cognitive tasks in terms of evoked and induced activities. There have been reports indicating that the neural activity reflecting cognitive information processing assessed in terms of evoked and induced activity, alpha and beta band activities, in particular, is associated with problem size in cognitive tasks (Grabner & De Smedt, 2011; Jost, Beinhoff, et al., 2004; Jost, Hennighausen, et al., 2004; Keil et al., 2006; Ku et al., 2010).

The excitatory post‐synaptic potentials (EPSPs) generated at the apical dendrite of the neurons are believed to be the source of the signals measured by MEG (Baillet et al., 2001). Since the current source caused by a single dendrite is estimated to be as little as 20 fA⸳m, which is too small to be detected by MEG, it is assumed that the accumulation of millions of EPSPs from a large ensemble of neurons can be measured using MEG (Hamalainen et al., 1993). Thus, the increase in cortical activity as assessed by MEG signals is not necessarily due to the increase of the number of neurons producing EPSPs or of the frequency of producing EPSPs in each neuron; it can also be caused by the resetting of the phase of spontaneous oscillations (Klimesch et al., 2007). Although debates have been held on the origin of evoked activities in regard to the relationship between evoked and induced activities, it is probable that both additive electromagnetic neural responses, which are independent of ongoing background oscillation, and the phase resetting of the ongoing background oscillation contribute to the generation of evoked activities (Sauseng et al., 2007). In this sense, the total electromagnetic cortical neuronal activity assessed in this study does not reflect the exact total investment of the neurons in the cerebral cortex to generate EPSPs. However, in addition to the additional generation of electromagnetic neural activities to the ongoing background oscillation, the phase resetting of the ongoing background oscillation is caused by information processing in neural networks (Canavier, 2015), so we believe that our estimates regarding the total electromagnetic cortical neuronal activity throughout the cognitive tasks approximated the total amount of cortical activity related to information processing throughout the tasks.

In this study, to reduce the signals unrelated to neural activities (i.e., noise) included in the measured MEG data, we limited the frequency range to 8–25 Hz, corresponding to the alpha (i.e., 8–13 Hz) and beta (i.e., 13–25 Hz) frequency bands. The saccadic spike potentials have been reported to be in the gamma (i.e., 30–100 Hz) frequency range (Yuval‐Greenberg & Deouell, 2011) and the frequencies below 5 Hz have been reported to be prone to movement artifacts (Tremmel et al., 2019). Therefore, we chose to analyze our MEG data in the 8–25 Hz frequency range to avoid overestimating the neural activity. On the one hand, this selection led to an underestimation of the total amount of cortical neuronal activity related to information processing because it eliminated signals in frequency bands lower and higher than alpha and beta, which are also related to cognitive function (Basar et al., 2001). On the other hand, it has been reported that alpha and beta neural activities reflect the problem size in cognitive tasks (Grabner & De Smedt, 2011; Jost, Beinhoff, et al., 2004; Jost, Hennighausen, et al., 2004; Keil et al., 2006; Ku et al., 2010), and that alpha neural activity reflects the attentional demand during information processing (Wei et al., 1998); therefore, limiting the frequency range of the MEG data to 8–25 Hz appears to be appropriate for approximating cortical neuronal activity, which reflects one of the major components of neural activity related to performing cognitive tasks, and avoiding an overestimation due to possible artifacts.

Together with an increase in fatigue levels, a decrease in motivation caused by performing cognitive tasks can work as a signal to suppress performance (Boksem et al., 2006). The negative association observed between the difference in the alteration of motivation levels and that in the total amount of cortical neuronal activity between the tasks suggests, for the first time, the possibility that the increase in total electromagnetic cortical neuronal activity caused by cognitive processes may be the source of information the brain depends on to create a sense of declined motivation for protection. Since no association was found between the difference in the alteration of the level of fatigue sensation and that in the total amount of cortical neuronal activity between tasks, the decreased motivation caused by performing cognitive tasks may be more primary information to suppress performance in fatigue than the increase of fatigue sensation; however, since we were not able to show that the correlation between the alteration of the level of motivation and that in the total amount of cortical neuronal activity was stronger than the correlation between the alteration of the level of fatigue sensation and that in the total amount of cortical neuronal activity for our present study, the idea that the decreased motivational level was more primary information to suppress performance rather than fatigue sensation is speculative. Further studies are needed on this point. Although it was difficult to determine the causal association between the difference in the alteration of motivation levels and that in the total amount of cortical neuronal activity between tasks in this study, it was unlikely that the decrease in motivation caused by performing cognitive tasks enhanced neuronal activity because it is difficult to suppose the situations in which cognitive processes are accelerated in the absence of motivation (i.e., the increase of neural activity to compensate for the decreased task performance would be caused through the increase of motivation).

There was no association between the alteration of the level of fatigue sensation and that of motivation in our present study. Together with the findings that the difference in the alteration of the total amount of cortical neuronal activity between the tasks was associated with motivation and not with fatigue sensation as discussed above, this seems to imply that the neural mechanism which causes the decrease of the motivation is not the same as that causes the increase of fatigue sensation in the situation of fatigue.

It is important to note that even if the increase in the total electromagnetic cortical neuronal activity caused by cognitive processes is the source of information the brain depends on to create a sense of declined motivation, as we suggest, the mechanisms by which the total activity is detected by our brain remain unknown. Although the neural mechanisms that directly assess the total neural activity caused in the brain may be discovered in the future, another possibility at present is that some kinds of humoral factors, such as cytokines, chemokines, inflammatory mediators, and reactive oxygen and nitrogen species, released by neurons in proportion to the amount of neural activity (Tanaka et al., 2013) can cause a decrease in motivation.

This study did have some limitations. First, neural activity during button pressing was not analyzed in this study because the noise caused by the finger movements to press the button can contaminate MEG signals, leading to an overestimation of the amount of neural activity. It is ideal to include the neural activity corresponding to the button pressing to assess the total electromagnetic neural activity caused by cognitive tasks; however, since we found that the difference in the alteration of motivation levels between tasks was negatively associated with that in the total amount of cortical activity, even after controlling for the difference in the number of button presses, the effect of excluding the neural activity corresponding to the button pressing on the relationship observed in this study seems to be minimal. Second, it is well known that the electromagnetic inverse problem (i.e., in this case, how to estimate neural current distributions from measured MEG signals) has no unique solution: we applied a minimum norm approach to estimate the neural current distributions in the cerebral cortex and the minimum norm estimation chooses the minimum energy solution to make the solution unique (Hamalainen & Ilmoniemi, 1994). However, since the minimum norm estimation is suggested for analyzing MEG data on a single trial level (Hauk, 2004) and therefore, our selection of this analytical method to assess the total electromagnetic cortical neuronal activity seems to be most appropriate.

In conclusion, we found a positive association between the decrease in motivation levels and the total amount of activity caused by the tasks. This finding suggests that the total amount of cortical neuronal activity is the source of the information for suppressing performance to avoid disrupting homeostasis and recovering from fatigue. These results could motivate further studies on the total electromagnetic cortical neuronal activity as the source of information utilized by biological alarms.

## CONFLICT OF INTEREST

The authors declare no conflicts of interest.

## AUTHOR CONTRIBUTIONS

**Akira Ishii:** conceptualization, methodology, investigation, formal analysis, data curation, writing‐original draft, writing‐review & editing; **Takashi Matsuo:** investigation; **Takahiro Yoshikawa:** supervision.

## Data Availability

The Ethics Committee of Osaka City University which approved the protocol of our present study does not allow public sharing of the original data. Code for the experimental task can be received by e‐mail upon reasonable request.
